# GIPC1 governed ferroptosis by regulating DECR1-modulating lipid homeostasis during dilated cardiomyopathy (DCM)

**DOI:** 10.1038/s41418-026-01679-9

**Published:** 2026-03-05

**Authors:** Nannan Tang, Ruxue Mu, He Wang, Jiaying Wu, Jie Zhang, Di Huang, Yannan Han, Wenjian Li, Yuqing Chen, Xiang Li, Yilin Sun, Zifeng Zhang, Jinlu Zuo, Ying Hu, Yanan Yin, Yang Qu, Jinping Liu, Lei Jiao, Xue Liu, Haihai Liang, Ning Wang, Yunlong Bai, Yan Liu, Bin Wang, Dan Zhao, Yu Liu, Baofeng Yang

**Affiliations:** 1 State Key Laboratory of Frigid Zone Cardiovascular Diseases (SKLFZCD), Department of Pharmacology (State Key Labratoray-Province Key Laboratories of Biomedicine-Pharmaceutics of China, Key Laboratory of Cardiovascular Research, Ministry of Education), College of Pharmacy, Harbin Medical University, Heilongjiang, 150081 China; 2 Department of Clinical Pharmacy, The Second Affiliated Hospital, Harbin Medical University, Heilongjiang, 150081 China; 3 Research Center for Pharmacoinformatics, College of Pharmacy, Harbin Medical University, Heilongjiang, 150081 China; 4 Department of Cardiovascular Ultrasound, Zhongnan Hospital of Wuhan University, Wuhan University, Wuhan, 430071 China; 5 Key Laboratory of Chronic disease Prevention and Control of Traditional Chinese Medicine of Guangdong Higher Education Institutes, School of Pharmaceutical Sciences, Guangzhou University of Chinese Medicine, Guangzhou, 510006 China; 6 College of Pharmacy, Hainan Medical University, Haikou, 571199 China

**Keywords:** Cardiomyopathies, Metabolic disorders

## Abstract

Dilated cardiomyopathy (DCM) was the most prevalent cardiomyopathy worldwide. Although ferroptosis has been implicated in cardiac pathogenesis, its regulatory mechanism in DCM remained poorly defined. In this study, we found that GIPC1 (GAIP/RGS19-interacting protein), a scaffolding protein, was significantly downregulated in cardiac tissues from DCM patients and doxorubicin (DOX)-induced DCM models. Integrated proteomic and lipidomic analysis revealed that cardiac-specific knockout of GIPC1 disrupted mitochondrial fatty acid metabolism, increased the abundance of polyunsaturated fatty acid-containing phospholipids (PUFA-PLs), and ultimately promoted ferroptosis in cardiomyocytes. Both in vitro and in vivo experiments demonstrated that GIPC1 deficiency exacerbated ferroptosis and cardiac dysfunction in DOX-induced cardiomyopathy, whereas GIPC1 overexpression conferred protection against ferroptosis in DOX-induced cardiomyopathy. Mechanistically, co-immunoprecipitation mass spectrometry (Co-IP/MS) and molecular docking demonstrated that GIPC1 interacted with mitochondrial 2,4-dienoyl-CoA reductase (DECR1) *via* its PDZ domain. Surface plasmon resonance (SPR) analysis further confirmed a high-affinity direct binding between GIPC1 and DECR1 (KD = 16.3 nM). Co-IP and immunofluorescence (IF) demonstrated that GIPC1 facilitated actin-dependent transport of DECR1 into mitochondria, thereby maintaining redox homeostasis and suppressing ferroptosis. Consistently, DECR1 overexpression rescued GIPC1 ablation-induced ferroptosis by balancing redox homeostasis. Together, these results demonstrated that GIPC1 reduced cardiomyocyte susceptibility to ferroptosis by promoting mitochondrial translocation of DECR1 and remodeling lipid homeostasis, highlighting GIPC1/DECR1 axis as a potential therapeutic strategy for DCM.

**Figure:**
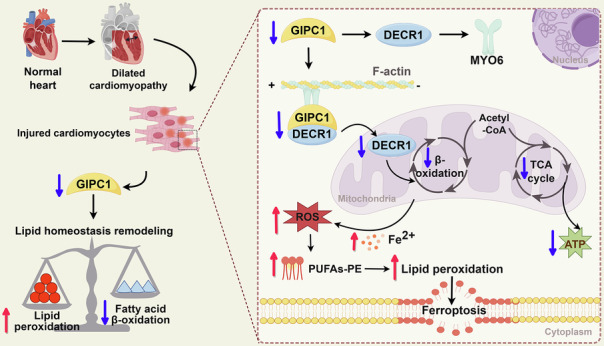
**A schematic model illustrating the pathogenic cascade triggered by GIPC1 deficiency during DCM**. In DCM, the expression level of GIPC1 was downregulated, thereby inhibiting actin-dependent transport of DECR1 into mitochondria, which remodeled lipid homeostasis and ultimately induced cardiomyocytes ferroptosis. Created with Figdraw.com.

**A schematic model illustrating the pathogenic cascade triggered by GIPC1 deficiency during DCM**. In DCM, the expression level of GIPC1 was downregulated, thereby inhibiting actin-dependent transport of DECR1 into mitochondria, which remodeled lipid homeostasis and ultimately induced cardiomyocytes ferroptosis. Created with Figdraw.com.

## Introduction

Dilated cardiomyopathy (DCM), the most prevalent form of cardiomyopathy worldwide, was typically manifested as heart failure, arrhythmia and even sudden cardiac death [1, 2]. Ferroptosis, an iron-dependent form of regulated cell death, has been implicated in the pathogenesis of various heart diseases [3, 4]. Nevertheless, the molecular mechanism underlying cardiomyocyte ferroptosis in DCM remained incompletely understood.

GIPC1 (GAIP/RGS19-interacting protein) acted as a scaffolding protein that played pivotal roles in cancer, neurological disorders, and cardiovascular diseases, primarily through PDZ domain-mediated protein interactions [5, 6]. Previous work from our group demonstrated that cardiac-specific knockout of GIPC1 in mice led to myocardial hypertrophy and fibrosis, largely attributable to reduced β1-adrenergic receptor protein stability [7]. Moreover, Hu et al., reported that the PDZ domain of GIPC1 directly interacted with the C-terminal ESKV motif of β1-adrenergic receptors, resulting in ERK1/2 activation in the human heart [8]. Sánchez et al., found that the interaction between transforming growth factor β receptor III (TGFβR3) and GIPC1 was essential for regulating epicardial cell proliferation and invasion during coronary vessel development [9]. Additionally, Sun et al., demonstrated that simvastatin prevented myocardial infarction-induced fibrosis and improved cardiac function through up-regulating TGFβR3/GIPC1 interaction and inhibiting ERK1/2/JNK pathway [10]. Despite these advances, the regulatory function of GIPC1 and its therapeutic potential in DCM remained unexplored.

Ferroptosis was a non-apoptotic cell death process driven by iron-dependent phospholipid (PL) peroxidation, which was regulated by the redox homeostasis, iron metabolism, mitochondrial dysfunction and impairment of the glutathione peroxidase 4 (GPX4)/glutathione (GSH) axis [11, 12]. The primary substrates for peroxidation were PLs containing polyunsaturated fatty acids (PUFAs) at the *sn2* position [13]. Emerging evidence has demonstrated that mitochondrial 2,4-dienoyl-CoA reductase (DECR1), a rate-limiting enzyme for β-oxidation of PUFAs, served as a vital regulator of ferroptosis in multiple cancers [14]. However, the current understanding of DECR1 in cardiac diseases is confined to its function in fatty acid β-oxidation (FAO) and mitochondrial biogenesis. Duan et al., reported that bone morphogenetic protein 9 upregulated mitochondrial DECR1 expression, thereby enhancing cardiac mitochondrial energy metabolism and attenuating myocardial infarction-induced injury [15]. Another study demonstrated that targeting DECR1 ameliorated diabetic cardiomyopathy by suppressing FAO *via* the PDK4/HDAC3 axis [16]. Yet, the involvement of DECR1 in regulating ferroptosis during the development and progression of cardiac disorders has not been reported.

In this study, we provided evidence that GIPC1 modulated lipid homeostasis by facilitating the mitochondrial translocation of DECR1, thereby protecting cardiomyocytes from ferroptosis in DCM. Our findings underscored a previously unrecognized role for GIPC1 in regulating lipid metabolism in DCM and suggested that targeting the GIPC1/DECR1 interaction may represent a promising therapeutic strategy for attenuating DCM progression.

## Results

### The expression level of GIPC1 protein was significantly decreased in the heart tissue of DCM patients and mice

**Fig. 1:**
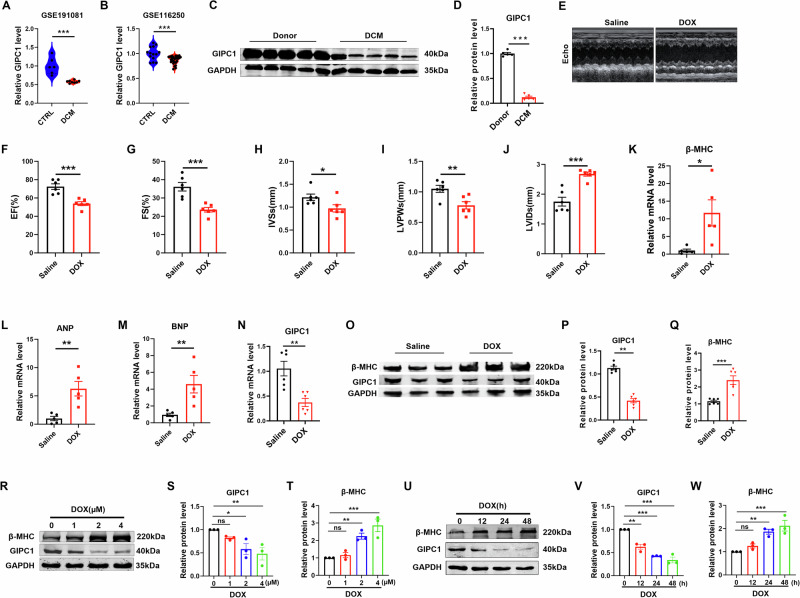
The expression level of GIPC1 decreased in the subjects with DCM. **A, B** The expression level of GIPC1 in the heart tissue of DCM patients and donors from GSE191081 and GSE116250. **C, D** Representative Western blot and quantitative analysis of GIPC1 protein in the heart tissue of patients with DCM. *n* = 5 per group. **E–J** Representative echocardiography and quantitative analysis in Saline and DOX group. *n* = 6 per group. **K–M** The mRNA levels of β-MHC, ANP and BNP in the heart tissue of the indicated groups. *n* = 5 per group. **N** The mRNA level of GIPC1 in the heart tissue of the indicated groups. *n* = 6 per group. **O–Q** Representative Western blot and quantitative analysis of GIPC1 and β-MHC protein in the heart tissue of DOX-induced DCM mice. *n* = 6 per group. **R–T** Representative Western blot and quantitative analysis of GIPC1 and β-MHC protein in NMCMs treatment with different concentrations of DOX (0, 1, 2, 4 µM) for 24 h. *n* = 3 per group. **U–W** Representative Western blot and quantitative analysis of GIPC1 and β-MHC protein in NMCMs with 2 µM DOX for 0, 12, 24, 48 h, respectively. *n* = 3 per group. All quantitative data were expressed as mean ± SEM, and statistical significance was analyzed using t-test, *P < 0.05, **P < 0.01, ***P < 0.001.

To verify whether the expression level of GIPC1 was altered in DCM patients, we analyzed 36 human samples from the GEO datasets and found that GIPC1 expression was significantly decreased in DCM patients (Fig. 1A, B). Strikingly, the level of GIPC1 protein was significantly decreased in the heart tissue of DCM patients compared with the donors (Fig. 1C, D). Then we constructed a mouse DCM model by administration of doxorubicin (DOX) for 4 weeks. Echocardiography showed that DOX-induced DCM mice exhibited cardiac dysfunction as evidenced by decreased ejection fraction (EF), fractional shortening (FS), the systolic septal thickness (IVSs), systolic left ventricular posterior wall thickness (LVPWs) and elevated systolic left ventricular internal dimensions (LVIDs) (Fig. 1E–J). The mRNA levels of the cardiac hypertrophic markers, including β-MHC, ANP and BNP were significantly increased in DOX-induced DCM mice (Fig. 1K–M). And the expression levels of GIPC1 mRNA and protein were strikingly decreased in DOX-induced DCM mice with the elevated expression level of β-MHC protein (Fig. 1N–Q). In vitro, the expression level of GIPC1 protein was decreased in a concentration and time dependent manner in neonatal mouse cardiomyocytes (NMCMs) after treatment with DOX. While the expression level of β-MHC protein was increased in NMCMs after  treatment with DOX (Fig. 1R–W). These results demonstrated that the expression level of GIPC1 protein was significantly decreased in the subjects with DCM.

### GIPC1 ablation impaired cardiac mitochondrial function via suppression of mitochondrial fatty acid metabolism

**Fig. 2:**
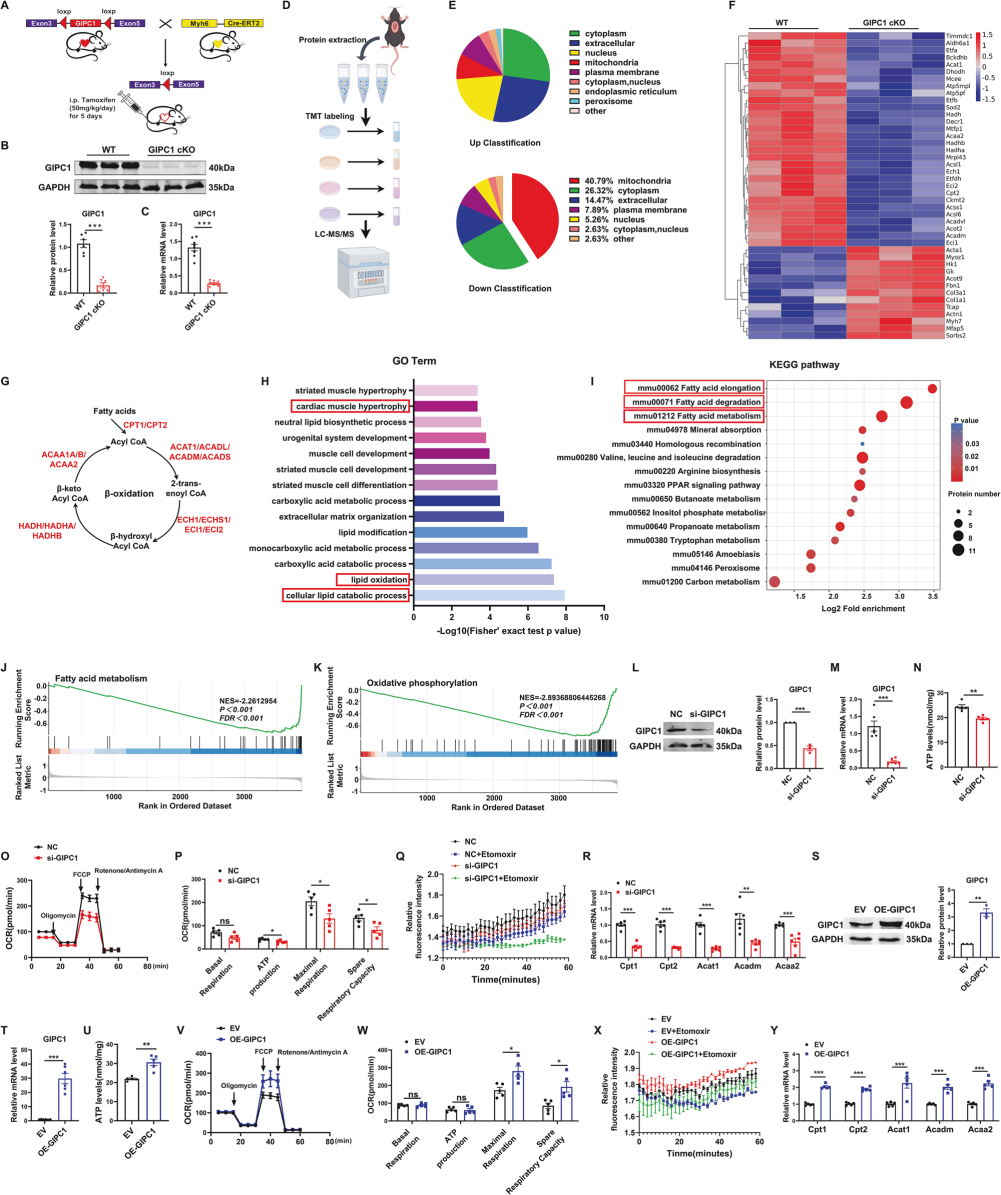
Knockout of GIPC1 downregulated mitochondrial fatty acid metabolism. **A** The schematic diagram for the generation of GIPC1 cKO mice. **B** Representative Western blot and quantitative analysis of GIPC1 protein in the heart tissues of GIPC1 cKO mice. *n* = 6 per group. **C** Real-time PCR analysis of GIPC1 mRNA level in the heart tissue of GIPC1 cKO mice. *n* = 8 per group. **D** The flow chart of the TMT-labeling proteomic analysis. *n* = 3 per group. Created with figdraw.com. **E** The pie chart indicating the subcellular localization of the differentially expression proteins in the heart of GIPC1 cKO mice. **F** The heatmap showing up-regulation and down-regulation proteins in GIPC1 cKO group heart. log2 (fold change) ≥ 1.2, *P* value < 0.05. **G** The schematic diagram of FAO process. Created with figdraw.com. **H** GO analysis of the differentially expressed proteins that were upregulated (red) and downregulated (blue) between WT and GIPC1 cKO groups. **I** KEGG pathway analysis of metabolic pathways enriched in the WT and GIPC1 cKO groups. **J, K** GSEA analysis of different proteins between the WT and GIPC1 cKO groups. **L** Representative Western blot and quantitative analysis of GIPC1 expression in NMCMs transfected with siRNA against GIPC1. *n* = 3 per group. **M** Real-time PCR analysis of GIPC1 mRNA level in NMCMs transfected with siRNA against GIPC1. *n* = 6 per group. **N** Measurement of ATP content in NMCMs transfected with siRNA against GIPC1. *n* = 5 per group. **O, P** Analysis of mitochondrialOCR, basal respiration, ATP production-coupled respiration, maximal respiration and spare respiratory capacity in NMCMs transfected with siRNA against GIPC1. *n* = 5 per group. **Q** Measurement of FAO content in NMCMs transfected with siRNA against GIPC1. *n* = 3 per group. **R** Real-time PCR analysis of mRNA levels of Cpt1, Cpt2, Acat1, Acadm and Acaa2 genes in NMCMs transfected with siRNA against GIPC1. *n* = 6 per group. **S** Representative Western blot and quantitative analysis of GIPC1 protein expression in NMCMs transfected with the GIPC1 overexpressing plasmid. *n* = 3 per group. **T** Real-time PCR analysis of GIPC1 mRNA level in NMCMs transfected with the GIPC1 overexpressing plasmid. *n* = 6 per group. **U** Measurement of ATP content in NMCMs transfected with the GIPC1 overexpressing plasmid. *n* = 5 per group. **V**, **W** Analysis and quantification of mitochondrial OCR, basal respiration, ATP production-coupled respiration, maximal respiration, and spare respiratory capacity in NMCMs transfected with the GIPC1 overexpressing plasmid. *n* = 5 per group. **X** Measurement of FAO content in NMCMs transfected with the GIPC1 overexpressing plasmid. *n* = 3 per group. **Y** Real-time PCR analysis of mRNA levels of Cpt1, Cpt2, Acat1, Acadm and Acaa2 genes in NMCMs transfected with the GIPC1 overexpressing plasmid. *n* = 5 per group. All quantitative data were expressed as mean ± SEM, and statistical significance was analyzed using t-test, *P < 0.05, **P < 0.01, ***P < 0.001.

To investigate the specific regulatory role of GIPC1 in the pathogenesis of DCM, we generated cardiac-specific conditional GIPC1 knockout (GIPC1 cKO) mice and validated by Western blot analysis and Real-time PCR (Fig. 2A–C). As previously reported, GIPC1 cKO mice exhibited cardiac dysfunction, along with cardiac remodeling manifested as interstitial fibrosis and hypertrophy (Supplementary Fig. 1A–K). Then we performed TMT-labeled quantitative proteomic analysis of the myocardial tissues of GIPC1 cKO mice (Fig. 2D). These results showed that a large proportion of the downregulated proteins was mainly distributed in mitochondria (Fig. 2E). These downregulated proteins were related to the process of mitochondrial fatty acid metabolism, including carnitine shuttle enzyme (Cpt2), key enzymes (Acaa2, Acadm, Acadvl, Hadha, Hadhb) and auxiliary enzymes (Eci1, Eci2, DECR1) (Fig. 2F, G). GO analysis and KEGG pathway showed that lipid metabolism-related processes were significantly enriched in the cardiac samples of GIPC1 cKO mice (Fig. 2H, I). GSEA further revealed a significant depletion of genes involved in the mitochondrial fatty acid metabolism and oxidative phosphorylation in GIPC1 cKO group compared to WT group (Fig. 2J, K). To further verify the regulatory effect of GIPC1 on fatty acid metabolism, we transfected the specific siRNA targeting GIPC1 into NMCMs (Fig. 2L, M). As expected, knockdown of GIPC1 decreased the ATP production (Fig. 2N) and inhibited mitochondrial oxygen consumption rate (OCR) (Fig. 2O, P). Furthermore, FAO analysis indicated that GIPC1 knockdown significantly decreased the level of FAO in NMCMs. Meanwhile, GIPC1 knockdown further downregulated the level of FAO induced by etomoxir, an inhibitor of CPT1 [17] (Fig. 2Q). And the mRNA levels of FAO-associated genes were significantly downregulated in NMCMs after GIPC1 knockdown (Fig. 2R). In contrast, we transfected the overexpression plasmid of GIPC1 into NMCMs (Fig. 2S, T). Overexpression of GIPC1 in NMCMs increased ATP production, OCR and FAO (Fig. 2U–X). The mRNA levels of FAO-associated genes were significantly upregulated in GIPC1 overexpressing NMCMs (Fig. 2Y). These results suggested that GIPC1 was an important determinant of mitochondrial fatty acid metabolism and cardiac mitochondrial function.

### GIPC1 deletion triggered ferroptosis in cardiomyocytes through disruption of intracellular lipid homeostasis

**Fig. 3:**
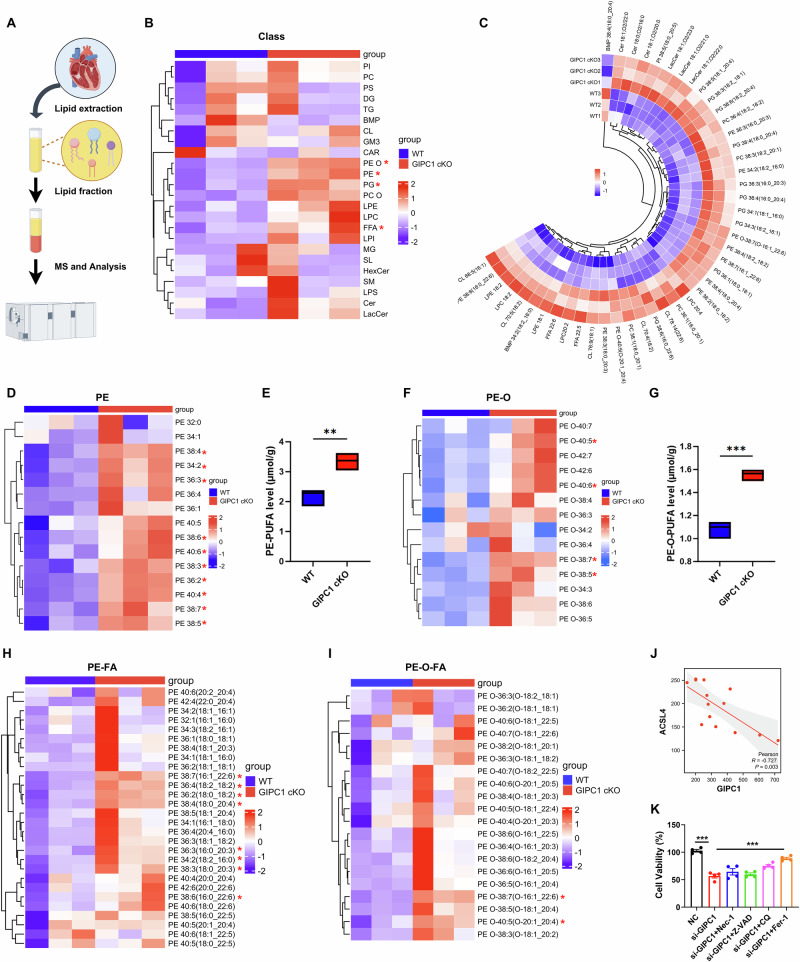
Knockout of GIPC1 upregulated PLs containing PUFAs. **A** The schematic diagram of non-targeted lipidomic analysis. *n* = 3 per group. Created with figdraw.com. **B** The heatmap showing the statistical analysis of the different lipid species between WT and GIPC1 cKO groups. **C** The heatmap showing the statistical significance of the different lipid species. Statistical significance was analyzed using the parametric t-test. **D, E** The heatmap and quantitative analysis of the content of PE-PUFAs. **F, G** The heatmap and quantitative analysis showing the content of PE-O-PUFA. **H, I** The heatmap showing the content of PE-FA and PE-O-FA. **J** Pearson analysis of the correlation between ACSL4 and GIPC1 in DCM patients. **K** The cell viability of NMCMs transfected with GIPC1 siRNA after treatment with different cell death inhibitors, Nec-1 (a necroptosis inhibitor), Z-VAD (an apoptosis inhibitor), CQ (an autophagy inhibitor) and Fer-1 (a ferroptosis inhibitor). *n* = 4 per group. All quantitative data were expressed as mean ± SEM, and statistical significance was analyzed using t-test and the one-way analysis of variance (ANOVA), *P < 0.05, **P < 0.01, ***P < 0.001.

Given the crucial role of GIPC1 in fatty acid metabolism, we performed the quantitative lipidomic analysis to evaluate the lipid profile in cardiac tissue of GIPC1 cKO mice (Fig. 3A). Compared with WT group, the GIPC1 cKO group exhibited significantly elevated levels of free fatty acid (FFA), phosphatidylglycerol (PG), phosphatidylethanolamine (PE), and plasmenyl-PE (PE-O) (Fig. 3B). Specifically, lipid species significantly upregulated in GIPC1 cKO mice were predominantly PLs containing PUFAs (Fig. 3C). Further subclass analysis revealed a marked increase in PUFA-containing PE and PE-O in GIPC1 cKO mice (Fig. 3D–I), along with a significant rise in PUFA-containing PG (Supplementary Fig. 2A). In contrast, other PUFA-containing PLs remained unchanged (Supplementary Fig. 2B–E). These findings indicated that GIPC1 ablation increased the abundance of PUFA-PLs, which may promote phospholipid peroxidation and ferroptosis. To validate this hypothesis, we analyzed 15 human DCM samples from the GEO datasets (GSE55296) and found that the expression level of GIPC1 protein was negatively correlated with the expression level of ACSL4 (Fig. 3J). To further confirm the role of GIPC1 knockdown in promoting ferroptosis, we treated NMCMs with various cell death inhibitors. The results revealed that GIPC1 knockdown significantly reduced the cell viability. This effect was rescued by Ferrostatin-1 (Fer-1), a ferroptosis inhibitor, but not by Necrostatin-1 (a necroptosis inhibitor), Z-VAD (an apoptosis inhibitor), or chloroquine (an autophagy inhibitor) [18] (Fig. 3K). These data demonstrated that GIPC1 deficiency promoted ferroptosis in cardiomyocytes by altering lipid homeostasis.

### GIPC1 conferred protection against ferroptosis by restoring the intracellular redox homeostasis in DOX-induced cardiomyocytes

**Fig. 4:**
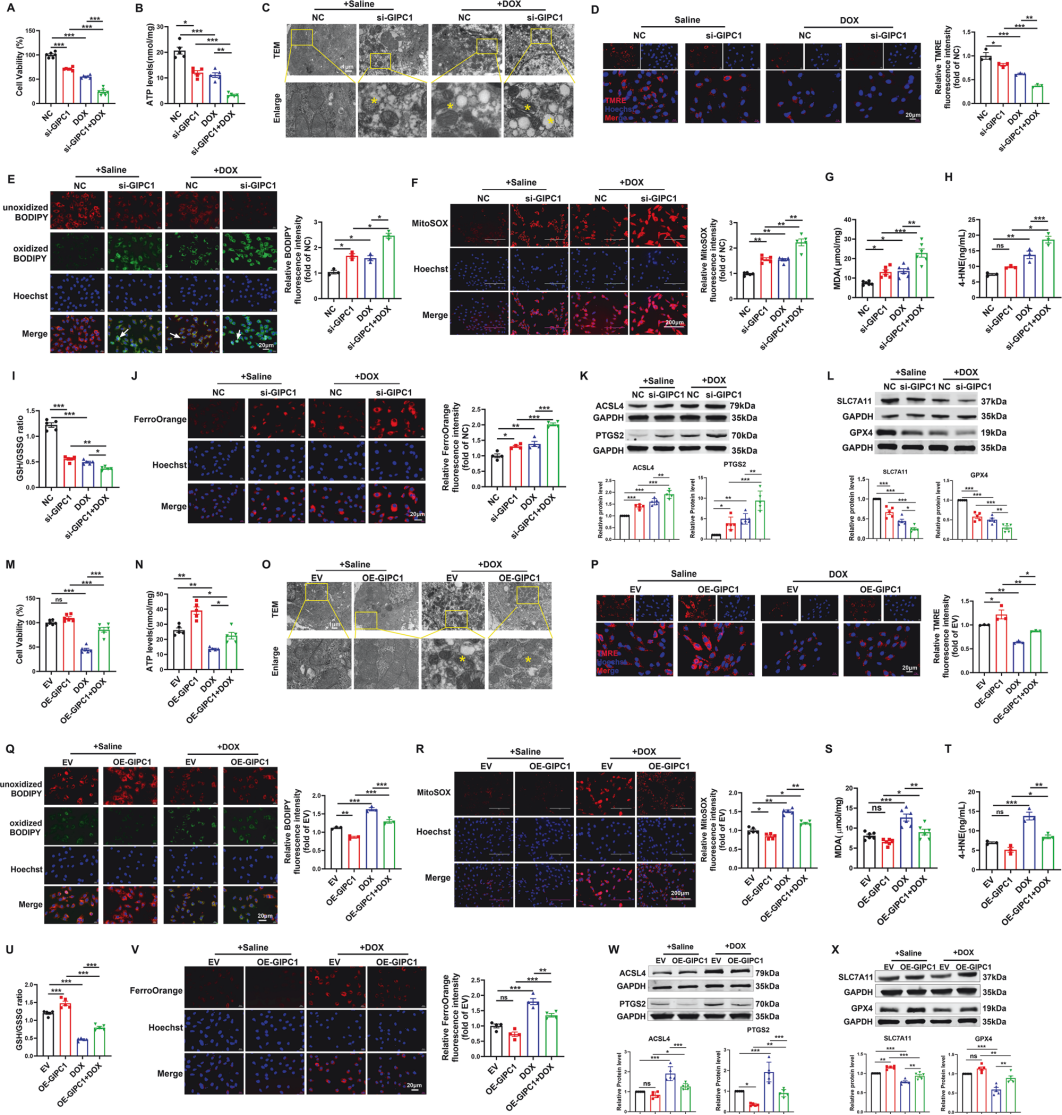
GIPC1 reversed DOX-induced ferroptosis in cardiomyocytes. **A**, **B** The quantitative analysis of cell viability (*n* = 6 per group) and ATP content (*n* = 5 per group) of NMCMs knockdown of GIPC1 treatment with DOX (2 μM) for 24 h. **C** Representative TEM images showing the ultrastructure of NMCMs knockdown of GIPC1 treatment with DOX. *: mitochondria with disrupted cristae. scale bar = 1 µm. *n* = 3 per group. **D** Representative images and quantitative analysis of mitochondrial membrane potential with TMRE staining in H9c2. scale bar = 20 μm. *n* = 3 per group. **E** Representative images and quantification of C11 BODIPY 581/591 staining in H9c2. scale bar = 20 μm. *n* = 3 per group. **F** Representative images and quantification of MitoSOX staining in H9c2. scale bar = 200 μm. *n* = 5 per group. **G–I** MDA content (*n* = 6 per group), 4-HNE level (*n* = 3 per group) and GSH/GSSG ratio (*n* = 5 per group) of NMCMs knockdown of GIPC1 treatment with DOX. **J** Representative images and quantitative analysis of cytoplasmic Fe^2+^ stained with FerroOrange in H9c2. scale bar = 20 μm. *n* = 4 per group. **K, L** Western blots and quantification of ACSL4, PTGS2, GPX4 and SLC7A11 protein levels in NMCMs. *n* = 5 per group. **M**, **N** The quantitative analysis of cell viability (*n* = 6 per group) and ATP content (*n* = 5 per group) of NMCMs overexpression of GIPC1 treatment with DOX (2 μM) for 24 h. **O** Representative TEM images showing the ultrastructure of NMCMs overexpression of GIPC1 treatment with DOX. *: mitochondria with disrupted cristae. scale bar = 1 µm. *n* = 3 per group. **P** Representative images and quantification of mitochondrial membrane potential with TMRE staining in H9c2. scale bar = 20 μm. *n* = 3 per group. **Q** Representative images and quantification of C11 BODIPY 581/591 staining in H9c2. scale bar = 20 μm. *n* = 3 per group. **R** Representative images and quantitative analysis of MitoSOX staining in H9c2. scale bar = 200 μm. *n* = 5 per group. **S–U** MDA content (*n* = 6 per group), 4-HNE level (*n* = 3 per group) and GSH/GSSG ratio *(n* = 5 per group) in NMCMs overexpression of GIPC1 treatment with DOX. **V** Representative images and quantitative analysis of cytoplasmic Fe^2+^ stained with FerroOrange in H9c2. scale bar = 20 μm. *n* = 4 per group. **W, X** Western blots and quantitative analysis of ACSL4, PTGS2, GPX4 and SLC7A11 protein levels in NMCMs. *n* = 5 per group. All quantitative data were expressed as mean ± SEM, and statistical significance was analyzed using the one-way analysis of variance (ANOVA), *P < 0.05, **P < 0.01, ***P < 0.001.

Subsequently, we investigated the regulatory role of GIPC1 in ferroptosis within DOX-induced cardiomyocytes. GIPC1 knockdown further reduced cell viability and ATP content in NMCMs compared with DOX group (Fig. 4A, B). DOX group displayed damaged mitochondria with a disarranged structure of cristae in NMCMs compared to the saline group. Knockdown of GIPC1 further damaged the mitochondrial structure compared to DOX group (Fig. 4C). Consistently, compared with DOX group, knockdown of GIPC1 further decreased mitochondrial membrane potential, and increased the level of oxidized BODIPY and MitoSOX (Fig. 4D–F). Additionally, GIPC1 knockdown further elevated the levels of Malondialdehyde (MDA) and 4-Hydroxynonenal (4-HNE), while decreased the GSH/GSSG ratio in DOX-treated NMCMs (Fig. 4G–I). FerroOrange staining confirmed that GIPC1 knockdown markedly enhanced intracellular free iron (Fe^2+^) accumulation compared with DOX group (Fig. 4J). Western blot showed that GIPC1 knockdown further upregulated the expression of the pro-ferroptotic markers ACSL4, PTGS2 and downregulated the expression of the ferroptotic suppressors GPX4 and SLC7A11 compared to the DOX group (Fig. 4K, L). Conversely, overexpression of GIPC1 attenuated DOX-induced mitochondrial lipid peroxidation and ferroptosis, leading to restore redox homeostasis and ameliorate cellular damage (Fig. 4M–X). Collectively, these results demonstrated that GIPC1 protected against ferroptosis by restoring intracellular redox homeostasis in DOX-induced cardiomyocyte injury.

### GIPC1 mitigated ferroptosis through modulation of the intracellular redox homeostasis in DOX-induced cardiomyopathy

**Fig. 5:**
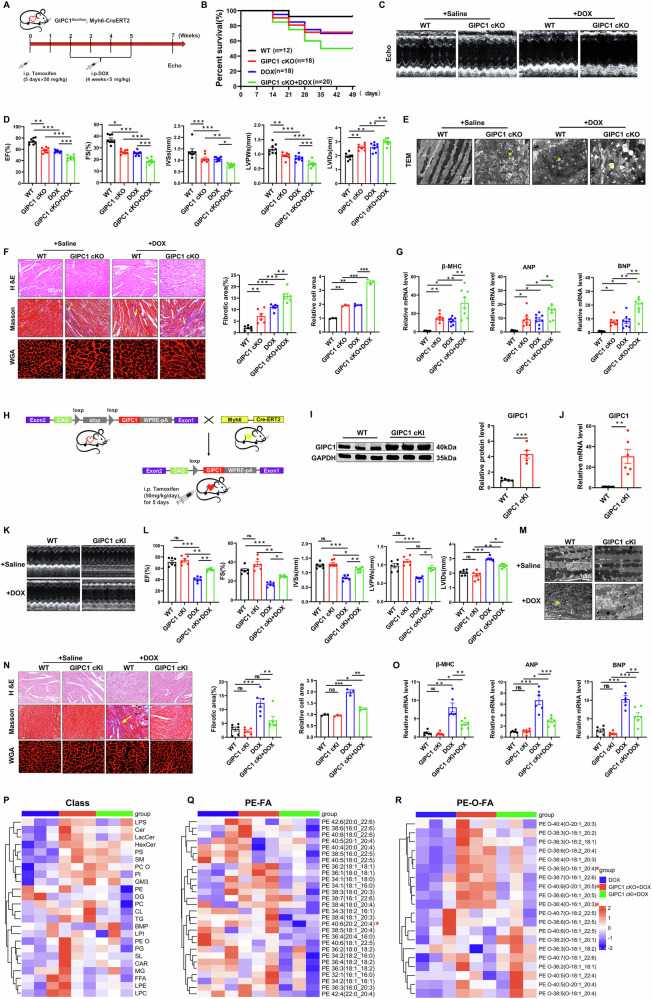
GIPC1 mitigated ferroptosis through remodeling the intracellular lipid homeostasis in DOX-induced cardiomyopathy. **A** The schematic diagram for the generation of DOX-induced DCM in mice. **B** Kaplan-Meier survival analysis showing survival rate of GIPC1 cKO mice after treatment with DOX. WT group: *n* = 12; GIPC1 cKO group: *n* = 18; DOX group: *n* = 18; DOX + GIPC1 cKO group: *n* = 20. **C, D** Echocardiographic analysis of GIPC1 cKO mice after treatment with DOX. *n* = 8 per group. **E** Representative TEM images showing the ultrastructure of the heart in GIPC1 cKO mice after treatment with DOX. *: mitochondria with disrupted cristae. scale bar = 1 µm. *n* = 3 per group. **F** The representative images of HE staining (*n* = 3 per group), Masson staining (*n* = 6 per group) and WGA staining (*n* = 3 per group) in the left ventricle of the indicated groups. Yellow arrow: fibrosis. scale bar = 50 µm. **G** The mRNA levels of β-MHC, ANP and BNP in the heart tissue of the indicated groups. *n* = 8 per group. **H** The schematic diagram for the generation of GIPC1 cKI mice. **I** Representative Western blot and quantitative analysis of GIPC1 protein in the heart tissue of GIPC1 cKI mice. *n* = 5 per group. **J** Real-time PCR analysis of GIPC1 mRNA level in the heart tissue of GIPC1 cKI mice. *n* = 6 per group. **K**, **L** Echocardiographic analysis of GIPC1 cKI group after treatment with DOX. *n* = 6 per group. **M** Representative TEM images showing the ultrastructure of the heart tissue in GIPC1 cKI mice after treatment with DOX. *: mitochondria with disrupted cristae. scale bar = 1 µm. *n* = 3 per group. **N** The representative images of HE staining (*n* = 3 per group), Masson staining (*n* = 6 per group) and WGA staining (*n* = 3 per group) in the left ventricle of the indicated groups. Yellow arrow: fibrosis. scale bar = 50 µm. **O** The mRNA levels of β-MHC, ANP and BNP in the heart tissue of the indicated groups. *n* = 6 per group. **P** The heatmap showing the statistical significance of the different lipid species. *n* = 3 per group. **Q** The heatmap showing the content of PE-FA. *n* = 3 per group. **R** The heatmap showing the content of PE-O-FA. *n* = 3 per group. All quantitative data were expressed as mean ± SEM, and statistical significance was analyzed using t-test and the one-way analysis of variance (ANOVA), *P < 0.05, **P < 0.01, ***P < 0.001.

Furthermore, we investigated the regulatory role of GIPC1 in ferroptosis under DOX-induced cardiomyopathy (Fig. 5A). Survival curve analysis revealed that only 50% of mice in the GIPC1 cKO+DOX group survived to 50 days, compared to 70% in the DOX group (Fig. 5B). Compared with DOX group, GIPC1 cKO+DOX group further deteriorated cardiac function (Fig. 5C, D). Electron microscopy showed that GIPC1 deletion exacerbated mitochondrial damage compared to the DOX group (Fig. 5E). Moreover, GIPC1 deletion aggravated myocardial structural remodeling, including interstitial fibrosis, and hypertrophy compared to the DOX group (Fig. 5F). The mRNA levels of the cardiac hypertrophic marker genes were also significantly increased in GIPC1 cKO+DOX group compared with DOX group (Fig. 5G). We subsequently generated cardiac-specific GIPC1 overexpression mice (GIPC1 cKI) (Fig. 5H) and further validated by Western blot analysis and Real-time PCR (Fig. 5I, J). Compared to the DOX group, GIPC1 overexpression ameliorated DOX-induced cardiac dysfunction, myocardial structural abnormalities as well as mitochondrial morphological damage (Fig. 5K–O). Lipidomic analysis revealed that GIPC1 deletion significantly increased the relative abundance of PUFA-containing PE species compared to DOX group, whereas GIPC1 overexpression counteracted these alterations (Fig. 5P–R). These results suggested that GIPC1 alleviated ferroptosis by restoring intracellular redox homeostasis, highlighting its potential as a therapeutic target for DCM.

### GIPC1 governed ferroptosis by facilitating the actin-based transport of DECR1 into mitochondria via its PDZ domain

**Fig. 6:**
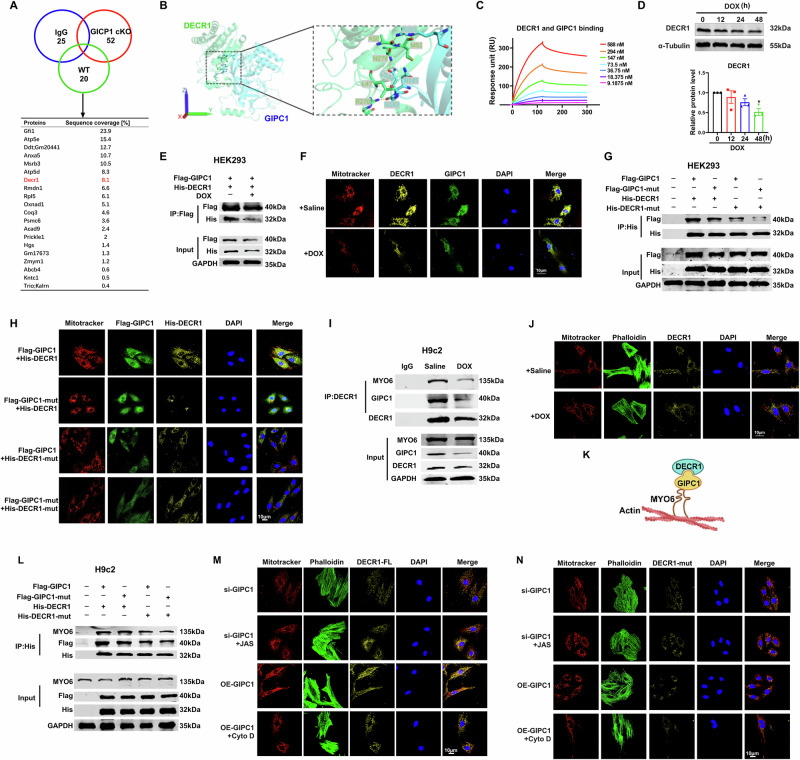
GIPC1 transported DECR1 into mitochondria through the actin-based manner. **A** The identification of the interacting proteins with GIPC1 by Co-IP/MS analysis. **B** The molecular docking showing the interaction between GIPC1 (cyan) and DECR1 (green). **C** SPR analysis showing the direct interaction between GIPC1 and DECR1. **D** Representative Western blot and quantitative analysis of DECR1 protein in NMCMs with 2 µM DOX for 0, 12, 24, 48 h, respectively. *n* = 3 per group. **E** Co-IP assay showing the interaction between Flag-GIPC1 and His-DECR1 after treatment with DOX for 24 h in HEK293 cells. *n* = 3 per group. **F** The locations of mitochondria (red), GIPC1 (green) and DECR1 (yellow) in NMCMs treated with DOX for 24 h were detected by IF. scale bar = 10 µm. **G** The interactions between Flag-GIPC1, Flag-GIPC1-mut, His-DECR1 and His-DECR1-mut detected by co-IP assay. *n* = 3 per group. **H** The locations of mitochondria (red), GIPC1 (green) and DECR1 (yellow) in H9c2 cells detected by IF. scale bar = 10 µm. *n* = 3 per group. **I** Co-IP assay showing the interaction of MYO6, GIPC1 and DECR1 after treatment with DOX in H9c2 cells. *n* = 3 per group. **J** The locations of mitochondria (red), DECR1 (yellow) and phalloidin (green) in H9c2 cells after treatment with DOX for 24 h by IF. scale bar = 10 µm. **K** The schematic diagram showing the interactions of GIPC1, DECR1 and MYO6. Created with figdraw.com. **L** The interactions between MYO6, Flag-GIPC1, Flag-GIPC1-mut, His-DECR1 and His-DECR1-mut detected by Co-IP assay. *n* = 3 per group. **M**, **N** The locations of mitochondria (red), DECR1 (yellow) and phalloidin (green) in H9c2 cells after transfected with siRNA or overexpression plasmid against GIPC1, and DECR1 plasmid as well as its mutation in the presence or absence of jasplakinolide/cytochalasin by IF. scale bar = 10 µm. *n* = 3 per group. All quantitative data were expressed as mean ± SEM, and statistical significance was analyzed using t-test, *P < 0.05, **P < 0.01, ***P < 0.001.

Subsequently, we performed co-immunoprecipitation mass spectrometry (Co-IP/MS) to elucidate the mechanism by which GIPC1 regulated ferroptosis. Compared with GIPC1 cKO mice, among the 20 interaction candidates enriched in the heart of WT mice, DECR1, a key regulator of ferroptosis [19], drew our attention (Fig. 6A). Molecular docking analysis predicted potential interactions between residues N182, G183, Q184, and R205 in the GIPC1 PDZ domain and residues L47, M51, R270, and N279 of DECR1 (Fig. 6B and Supplementary Fig. 3A–E). Surface plasmon resonance (SPR) analysis further confirmed a high-affinity direct binding between GIPC1 and DECR1 (KD = 16.3 nM) (Fig. 6C). Western blot analysis showed that the expression level of DECR1 was decreased in a time-dependent manner in cardiomyocytes treated with DOX (Fig. 6D). Co-IP and IF assays verified that GIPC1 interacted with DECR1 and co-localized within mitochondria, an effect that was abolished by DOX treatment (Fig. 6E, F). While mutation of these binding sites to alanine (A) attenuated the GIPC1/DECR1 interaction as well as their mitochondrial localization (Fig. 6G, H). Co-IP assays further revealed that GIPC1/DECR1 complex interacts with Myosin VI (MYO6), an actin-based motor protein [20], and these interactions were weakened by DOX treatment (Fig. 6I). IF analysis suggested that the actin-dependent mitochondrial transport of DECR1 into mitochondria was significantly impaired after treatment with DOX (Fig. 6J). Moreover, GIPC1 mutation and DECR1 mutation abolished the interaction with MYO6 (Fig. 6K, L). Knockdown of GIPC1 impaired actin-dependent mitochondrial transport of DECR1, which was rescued by jasplakinolide, an actin stabilizer [21]. Conversely, GIPC1 overexpression enhanced DECR1 transport into mitochondria, which was reversed by cytochalasin, an actin depolymerization inhibitor [22]. Nevertheless, these regulatory effects were diminished in DECR1 mutant group (Fig. 6M, N). Collectively, these findings indicated that GIPC1 mediated actin-based mitochondrial targeting of DECR1, and this process was impaired in DOX-induced cardiomyopathy.

### Overexpression of DECR1 alleviated GIPC1 ablation-induced ferroptosis by maintaining the intracellular lipid homeostasis

**Fig. 7:**
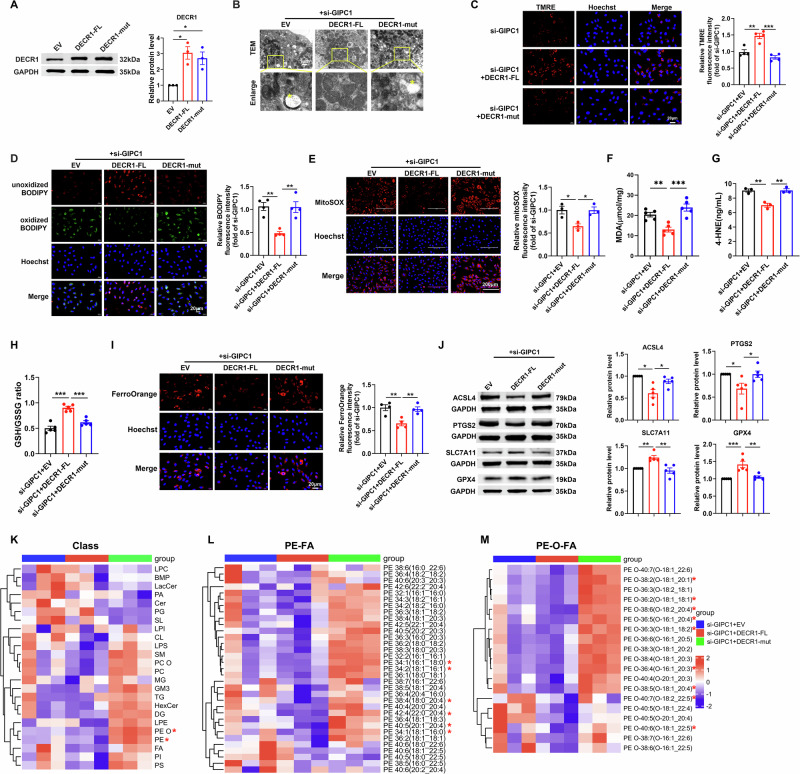
DECR1 overexpression reversed GIPC1 deletion-induced ferroptosis. **A** Representative Western blot and quantification of DECR1 protein expression in NMCMs transfected with the DECR1 overexpressing or mutation plasmid. *n* = 3 per group. **B** Representative TEM images showing the ultrastructure of NMCMs. *: mitochondria with disrupted cristae. scale bar = 1 µm. *n* = 3 per group. **C** Representative images and quantitative analysis of mitochondrial membrane potential with TMRE staining in H9c2. scale bar = 20 μm. *n* = 4 per group. **D** Representative images and quantitative analysis of C11 BODIPY 581/591 staining in H9c2. scale bar = 20 μm. *n* = 4 per group. **E** Representative images and quantitative analysis of MitoSOX staining in H9c2. scale bar = 200 μm. *n* = 3 per group. **F–H** MDA content (*n* = 5 per group), 4-HNE level (*n* = 3 per group) and GSH/GSSG ratio in NMCMs (*n* = 5 per group). **I** Representative images and quantitative analysis of cytoplasmic Fe^2+^ stained with FerroOrange in H9c2. scale bar = 20 μm. *n* = 4 per group. **J** Western blots and quantitative analysis of ACSL4, PTGS2, GPX4 and SLC7A11 protein levels in NMCMs. *n* = 5 per group. **K–M**  The heatmap showing the statistical significance of the different lipid species in the indicated groups. *n* = 3 per group. All quantitative data were expressed as mean ± SEM, and statistical significance was analyzed using the one-way analysis of variance (ANOVA), *P < 0.05, **P < 0.01, ***P < 0.001.

To investigate the role of DECR1 in GIPC1 ablation-induced ferroptosis, we transfected cardiomyocytes with either a full-length DECR1 overexpression plasmid (DECR1-FL) or a loss-of-function mutant plasmid (DECR1-mut), respectively (Fig. 7A). Compared to the GIPC1 knockdown group, DECR1-FL overexpression attenuated mitochondrial damage and increased mitochondrial membrane potential, whereas the DECR1 mutant had no such effects (Fig. 7B, C). Accordingly, DECR1-FL overexpression, but not the mutant, significantly reduced levels of oxidized BODIPY, MitoSOX, MDA and 4-HNE, while increased the GSH/GSSG ratio relative to GIPC1 knockdown cells (Fig. 7D–H). Additionally, overexpression of DECR1 markedly reduced intracellular Fe^2+^ accumulation induced by GIPC1 deletion (Fig. 7I). Western blot analysis indicated that DECR1 overexpression upregulated the key ferroptotic suppressors and downregulated the pro-ferroptotic markers compared to GIPC1 knockdown group, while the DECR1 mutant had no significant effect on these markers (Fig. 7J). Lipidomic profiling further demonstrated that DECR1 overexpression significantly reduced the relative abundance of PUFA-containing PE species in comparison to the GIPC1 deletion group, an effect not observed within the mutant DECR1 (Fig. 7K–M). Taken together, these results indicated that DECR1 reversed GIPC1 deficiency-induced ferroptosis by restoring lipid homeostasis.

## Discussion

This study demonstrated that GIPC1 maintained intracellular lipid homeostasis by facilitating the actin-based transport of DECR1 into mitochondria, thereby protecting cardiomyocytes against ferroptosis in DOX-induced DCM. Collectively, these findings not only elucidated a novel protective function of GIPC1 in DCM pathogenesis, but also supported its therapeutic potential as a molecular target for intervention.

DOX was a widely used chemotherapeutic agent whose application was limited by dose-dependent cardiotoxicity, including DCM and heart failure [23, 24]. Growing evidence indicated that ferroptosis significantly contributed to DOX-induced cardiomyopathy. Specifically, DOX induced iron overload through upregulation of the transferrin receptor and inhibition of ferritin, leading to excessive ROS generation [25]. DOX suppressed both cytosolic and mitochondrial GPX4, resulting in uncontrolled lipid peroxidation and ferroptosis [26]. Cui et al., reported that protosappanin A protected against DOX-induced myocardial injury and cardiac dysfunction by targeting the ACSL4/FTH1 axis to suppress ferroptosis [27]. Similarly, Wang et al., demonstrated that PRMT4 accelerated ferroptosis in DOX-induced cardiomyopathy by inhibiting the Nrf2/GPX4 pathway [28]. These studies collectively highlighted ferroptosis inhibition as a promising therapeutic strategy for alleviating DOX-induced cardiotoxicity. However, to date, dexrazoxane remained the only FDA-approved iron chelator for cardioprotection in DOX-treated patients [29, 30]. In this study, we identified GIPC1 as a suppressor of ferroptosis through maintaining intracellular lipid homeostasis by facilitating the actin-based transport of DECR1 into mitochondria in DOX-induced DCM, which underscored the therapeutic potential of targeting GIPC1 in DOX-induced cardiotoxicity.

Ferroptosis was an iron-dependent form of regulated cell death driven by the accumulation of phospholipid peroxides, which was regulated by multiple cellular metabolic pathways [31]. Liang et al., reported that PUFA-containing phospholipids (PUFA-PLs) played a central role in propagating lipid peroxidation, a pivotal event in ferroptosis execution [13]. Kagan et al., further identified PEs, especially those esterified with arachidonoyl (AA, C20:4) or adrenoyl (AdA, C22:6) chains, as the phospholipids most susceptible to peroxidation. Importantly, inhibiting the esterification of AA or AdA into PE through genetic or pharmacological targeting of ACSL4 constituted a specific anti-ferroptotic rescue pathway [32]. Our lipidomic analysis revealed that GIPC1 deletion resulted in significantly elevated levels of PG, PE, and PE-O species. Specifically, GIPC1 knockout increased the abundance of specific AA/AdA-esterified PE species, including PE (16:1_22:6), PE (18:0_20:4), PE (16:0_22:6), PE-O (16:1_22:6) and PE-O (20:1_20:4) compared to the WT group. Furthermore, GIPC1 deficiency enhanced the accumulation of PUFA-containing PE species following DOX treatment, an effect that was rescued by DECR1 overexpression in cardiomyocytes. These results established GIPC1 as a critical regulator of phospholipid homeostasis through its interaction with DECR1, ultimately modulating ferroptosis in DOX-induced cardiomyopathy.

Fatty acid β-oxidation (FAO) played an essential role in energy production and was closely linked to ferroptosis regulation [33]. This cyclic process shortened fatty acyl chains and generated NADH, FADH_2_, and acetyl-CoA [34]. FAO served a dual role in regulation of ferroptosis. On one hand, FAO-derived reactive oxygen species (ROS) can promote lipid peroxidation and ferroptosis. Guo et al., demonstrated that CCN1 acted as a key regulator of ferroptosis thereby enhancing ROS production through abnormal FAO activation in lung cancer [35]. Similarly, Yang et al., demonstrated that BRD4, in complex with HMGB2, bound to super-enhancers at the promoter regions of fatty acid metabolism genes HADH, ACSL1, and ACAA2, thereby promoting fatty acid metabolism and sustaining ferroptosis sensitivity [36]. On the other hand, FAO also prevented ferroptosis by balancing redox homeostasis. Miess et al., found that impaired FAO increased the susceptibility of renal cell carcinoma to ferroptosis [37]. Chen et al., showed that FABP4 regulated high glucose-induced ferroptosis by inhibiting FAO in HK2 cells [38]. Furthermore, Kalucka et al., demonstrated that quiescent endothelial cells depended on FAO to enhance NADPH regeneration, thereby supporting redox balance and vasculoprotection [39]. In our study, we observed that GIPC1 deletion suppressed mitochondrial FAO, leading to the accumulation of PUFA-PE, a process that typically triggers lipid peroxidation and ferroptosis during DCM.

GIPC1 consisted of GIPC homology 1 (GH1) domain, PDZ domain and GH2 domain. The regions around the GH1 and GH2 domains of GIPC1 were involved in dimerization and interaction with MYO6, respectively [40]. GIPC1 functioned as an adaptor protein that interacted with cargo molecules through its PDZ domain, while MYO6 acted as actin-based motor protein that moved toward the minus end of actin filaments [41], which was significantly implicated in cargo trafficking, signal transduction, and cargo recycling. Canon et al., revealed that GIPC1 bound with MYO6, released its autoinhibition and triggered proximal dimerization [42]. Previous studies showed that GIPC1-MYO6 complex was essential for BDNF-TrkB-mediated synaptic plasticity in the hippocampus [43]. Naccache et al., showed that binding of internalized receptors to the PDZ domain of GIPC/synectin recruited MYO6 to endocytic vesicles [20]. Furthermore, GIPC1 facilitated actin-based transport of Drp1 to perinuclear mitochondria, thereby promoting mitochondrial fission and enhancing proliferative and migratory capacities in cancer cells [44]. In this study, using Co-IP/MS, Co-IP, SPR, and IF assays, we confirmed that GIPC1 directly interacted with DECR1 with a high-affinity binding (KD = 16.3 nM). Specifically, the PDZ domain of GIPC1 loaded DECR1 as a cargo for MYO6-driven actin-based transport into mitochondria.

DECR1, an auxiliary enzyme in β-oxidation, metabolized unsaturated enoyl-CoA esters with double bonds at both even- and odd-numbered positions [45]. DECR1 has been increasingly recognized as a regulator of ferroptosis through remodeling phospholipid composition and redox homeostasis [46]. For instance, DECR1 helped maintain redox balance by regulating the ratio of saturated to unsaturated phospholipids in castration-resistant prostate cancer [47]. Wu et al., reported that bufalin induced ferroptosis in breast cancer *via* the DECR1-SLC7A11 axis, accompanied by elevated MDA and iron levels [48]. Nassar et al., showed that DECR1 knockdown selectively inhibited PUFA β-oxidation, leading to oxidative stress and lipid peroxidation-induced ferroptosis in prostate cancer [14]. Zhao et al., revealed that METTL3-mediated m^6^A modification promoted YTHDF3-dependent DECR1 degradation, resulting in PUFA accumulation and ferroptosis [49]. Consistent with these findings, Yang et al., confirmed that DECR1 knockdown elevated classic ferroptosis markers, including intracellular and mitochondrial ROS, Fe^2+^, and MDA [50]. Consistently, our results demonstrated that overexpression of DECR1 significantly reduced the relative abundance of PUFA-containing PE species in GIPC1-knockdown cardiomyocytes, thereby attenuating GIPC1 deficiency-induced ferroptosis through balancing redox homeostasis. Furthermore, this study also identified key residues in DECR1 binding with GIPC1, including L47, M51, R270, and N279. Mutation of these sites to alanine (A) abolished mitochondrial targeting of DECR1 by GIPC1, ultimately preventing the rescue of both PUFA-PE accumulation and ferroptosis in GIPC1-deletion cardiomyocytes.

In summary, this study established GIPC1 as a key regulator of lipid homeostasis *via* interaction with DECR1 through its PDZ domain, thereby protecting against ferroptosis in DCM. These findings provided that targeting the GIPC1/DECR1 complex may offer a novel preventive strategy for preventing and treating DCM.

## Materials and methods

### Human heart samples

The heart samples were collected from 5 patients with DCM and 5 healthy subjects. All procedures complied with the Declaration of Helsinki and relevant national guidelines for the use of human tissues. All patients themselves or their legal representatives voluntarily signed the informed consent to participate in the study and permitted the use of their heart tissue.

### Animal models

The C57BL/6 mice (6-8 weeks old, 22-25 g) were maintained in a temperature-controlled facility with 12 h light/dark cycle at 23 ± 3 °C and 30-70% relative humidity. To establish the DCM model, mice were intraperitoneally injected with DOX (E2516, Selleck, USA) at a dose of 5 mg/kg per week for 4 weeks (a cumulative dose of 20 mg/kg).

### Generation of conditional GIPC1 knockout mice (GIPC1 cKO mice)

GIPC1 cKO mice were generated by VIEWSOUD BIOTECH (Beijing, China). Briefly, GIPC1 cKO mice were established using the CRISPR/Cas9 system. The exon 3 coding sequence (CDS) region of mouse GIPC1 gene was cloned and flanked by two LoxP sites. Furthermore, two single-guide RNAs (gRNA1: AGGCTGGGTGGGAGTCTAGGG and gRNA2: CTCCTGGAAGTCGGGTGTGGG) targeting two locations in the CDS region were designed and synthesized. The primers used for verifying the floxed allele function as following. The sequences of primer F1/R1 were 5’-GGCAGAGATTCAAGTAGAGG-3’ (F1), 5’-CCAAGGAGAGGTCCACAT-3’ (R1). The sequences of primer F2/R2 were 5’-CACTGGGATGACCTTGAG-3’ (F2), 5’-GCTAATGGCTCCTGGAAG-3’ (R2), respectively. The founder mouse was mated with C57BL/6 J female mice to obtain GIPC1^flox/flox^ mice, which were crossed with MEM-Cre transgenic mice (α-MHC-MerCreMer) (Cyagen Biosciences; China) to produce GIPC1^flox/flox^/MEM-Cre mice. Furthermore, the GIPC1^flox/flox^/MEM-Cre mice at 6 weeks of age were injected intraperitoneally with tamoxifen (50 mg/kg) (Sigma-Aldrich, USA) daily for 5 consecutive days to generate GIPC1 cKO mice. GIPC1^flox/flox^ mice without tamoxifen treatment were served as control group.

### Generation of cardiac-specific GIPC1-TG mice (GIPC1 cKI mice)

GIPC1 cKI mice were constructed by Shanghai Model Organisms (China). Briefly, the cDNA sequence (GGGGACACACTAAGGGAGCTTGG) was inserted into pCAG-loxP-CAT-loxp-lacZ by replacing the LacZ gene to generate the plasmid of pCAG-CAT-mGIPC1 containing the CAG promoter and the LoxP-flanked CAT gene. The vector was microinjected into fertilized murine embryos to generate the transgenic founders. The primers used for verifying the floxed allele function as following. The sequences of primer F1/R1 were 5’-TCAGATTCTTTTATAGGGGACACA-3’ (F1) and 5’-TAAAGGCCACTCAATGCTCACTAA-3’ (R1). The sequences of primer F2/R2 were 5’-GGCAAGGCAGGCGGCGATGAG-3’ (F2) and 5’-GGGCAGCGCAAAGAGGTGGAAGTG-3’ (R2). The generated CAG-CAT-mGIPC1 mice were bred with α-MHC-MerCreMer mice to generate CAG-CAT-mGIPC1/α-MHC-MerCreMer double-transgenic mice. The CAG-CAT-mGIPC1/α-MHC-MerCreMer mice at 6 weeks of age were injected intraperitoneally with 50 mg/kg tamoxifen (Sigma-Aldrich, USA) daily for five consecutive days to generate GIPC1 cKI mice. The CAG-CAT-GIPC1 mice without tamoxifen administration served as the control group.

### Echocardiography

Two-dimensional (2D) guided M-mode echocardiography was performed using the Vevo2100 Imaging System (VisualSonics, Toronto, Canada). Echocardiographic assessment of cardiac function in conscious and anesthetized mice was recorded from parasternal short-axis views of the left ventricular (LV). Collected parameters included ejection fraction (EF), fractional shortening (FS), the systolic septal thickness (IVSs) systolic left ventricular posterior wall thickness (LVPWs) and the systolic left ventricular internal dimensions (LVIDs). The EF and FS were automatically calculated and expressed as percentages by the system.

### Transmission electron microscopy (TEM)

The left ventricle was removed and dissected into 1-2 mm^3^ pieces and fixed in 2.5% glutaraldehyde at 4 °C overnight. Digested NMCMs were washed with phosphate-buffered saline (PBS) and fixed in 2.5% glutaraldehyde at 4 °C overnight. The samples were rinsed and dehydrated in graded alcohol. Then the samples were embedded in Epon, cut into ultrathin 90 nm-thick sections and finally counterstained with the uranyl acetate and lead citrate. The ultrastructural images were obtained with a JEOL 1200 electron microscope (JEOL Ltd, Tokyo, Japan).

### Hematoxylin-eosin staining (HE)

The samples were fixed in 4% paraformaldehyde, embedded in paraffin, and cut cross-sectionally into 5-μm-thick sections along the center of the fibrotic scar. Staining was performed according to the instructions of the HE staining kit (G1120, Solarbio, China). The sections were imaged at × 200 magnification by bright-field microscopy (IX71 Olympus, Japan).

### Masson staining

As mentioned above, the sections were stained with Masson’s trichrome (G1340, Solarbio, China) to evaluate collagen deposition. Cardiac fibrosis was determined by calculating the ratio of collagen surface areas stained blue to the myocardial surface area. All quantitative evaluations were assessed by ImageJ software (NIH, USA).

### Wheat germ agglutinin staining (WGA)

Dissected tissues were stored in 30% sucrose/PBS at 4 °C for 24 h. Thereafter, the tissues were embedded in OCT compound and sectioned at a thickness of 7 micrometers. The surface area of cardiomyocytes was quantified from heart sections that had been stained with wheat germ agglutinin, Alexa Fluor 594 conjugate (W11262, Invitrogen, USA). All quantitative evaluations were assessed by Image J software (Media Cybernetics, USA).

### Isolation of Neonatal mouse cardiomyocytes (NMCMs)

NMCMs were isolated from 1-3-day-old neonatal mice and washed in 1×PBS and then digested in 0.05% pancreatin (BL1093A, Biosharp, China) at 4 °C overnight. Then the tissues were digested in DMEM (Gibco, USA) containing 0.8 mg/mL collagenase II (17101015, Gibco, USA) on a rotary shaker at 37 °C. The obtained cells were centrifuged at 1000 rpm for 5 min and resuspended by DMEM containing 10% fetal bovine serum (FBS) (Biological Industries, Israel) and 1% penicillin/streptomycin (BL505A, Biosharp, China). The cells were incubated at 37 °C in a humidified atmosphere of 5% CO_2_ and 95% air for 2 h to remove cardiac fibroblasts.

### Cell culture and transfection

HEK293 and H9c2 cells were purchased from BNCC (Beijing, China) and cultured in DMEM supplemented with 10% FBS and 1% penicillin-streptomycin at 37 °C in a humidified atmosphere of 5% CO₂. The coding sequences for human GIPC1 and DECR1 were amplified by PCR and cloned into expression vectors to generate Flag-GIPC1 and His-DECR1 expression plasmids, respectively (GeneChem, Shanghai, China). Site-directed mutagenesis was performed to generate GIPC1 mutants (N182A/G183A/Q184A/R205A) and DECR1 mutants (L47A/M51A/R270A/N279A). All constructs were verified by DNA sequencing (Hanyin, Shanghai, China). Plasmid sequences are provided in Supplementary Table 1. After the cells reached 60-70% confluence, Flag-GIPC1 and His-DECR1 expression plasmids were transfected using Lipofectamine™ 2000 (Invitrogen, USA), according to the manufacturer's instructions. For knockdown experiments, cells were transfected with GIPC1-specific siRNA (Genepharma, Suzhou, China) or negative control siRNA using the X-tremeGENE transfection reagent (Roche, Switzerland). siRNA sequences are listed in Supplementary Table 2.

### Western blotting

The total extraction from the cells and heart tissues was lysed with RIPA lysis buffer (P0013B, Beyotime Biotechnology, China) containing protease inhibitors. The BCA protein assay kit (P0011, Beyotime Biotechnology, China) was used to determine the protein concentration. Then the proteins were separated by SDS-PAGE, and then transferred to nitrocellulose filter (NC) membrane which were incubated with the following antibodies (Supplementary Table 3). The membrane was scanned with an Odyssey CLx System (LI-COR Biosciences). Western blot bands were quantified using QuantityOne software by measuring the band intensity (area ×OD) normalized to an internal control.

### Real-Time PCR

Total RNA samples were extracted by Trizol reagent (Invitrogen, Carlsbad, America) according to manufacturer’s protocols. The RNA levels were determined with SYBR Green PCR Master Mix (Roche, Switzerland) on a QuantStudioTM 6 Flex real-time PCR system (4306737, Applied Biosystems, USA) with GAPDH as an internal control. The relative RNA level was analyzed by using 2^-ΔΔct^ method. The sequences of the primers used in this experiment are listed in Supplementary Table 4.

### Immunostaining

Samples were washed 3 times and stained with 100 nM MitoTracke Deep Red CMXRos (M22426, Invitrogen, USA) at 37 °C for 30 min. Then, the samples were washed and fixed with 4% paraformaldehyde for 15 min at room temperature. 0.1% Triton X-100 was added for 30 min at room temperature and blocked with 10% normal goat serum at 37 °C for 2 h. Samples were incubated with primary antibodies at 4 °C overnight. After washing with PBS, samples were incubated with the secondary antibody at room temperature for 1 h without light. And the nuclei were labeled with DAPI (P0131, Beyotime Biotechnology, China) for 15 min at room temperature without light. Images were obtained by a laser scanning confocal microscope (Handbuch LSM 880, Carl Zeiss, Germany). The antibodies used for immunostaining assay were as follow. GIPC1(sc-271822, Santa Cruz Biotechnology, CA, 1:100 dilution), DECR1 (bs-5056R, Bioss, China, 1:200 dilution), Alexa Fluor™ 488-conjugated mouse secondary antibody (A-11001, Invitrogen, USA, 1:500 dilution), Alexa Fluor™ 594-conjugated Rabbit secondary antibody (A-11002, Invitrogen, USA).

### Phalloidin staining

After treatment with 20 nM jasplakinolide (sc-202191, Santa Cruz Biotechnology, CA) or 200 nM cytochalasin-d (PHZ1063, Invitrogen, USA), samples were fixed with 4% paraformaldehyde and stained with FITC- phalloidin (RM02836, ABclonal, China, 1:200 dilution) for 30 min in dark. Images were obtained by a laser scanning confocal microscope (Handbuch LSM 880, Carl Zeiss, Germany).

### Cell counting kit 8 (CCK8) assay

Cell viability was evaluated using the CCK-8 assay (HY-K0301, MedChemExpress, USA) according to the manufacturer’s protocol. Briefly, NMCMs were seeded in 96-well plates and incubated overnight. The next day, cells were transfected with GIPC1 siRNA or GIPC1-overexpressing plasmid for 48 h and were treated with 2 μM DOX for 24 h or inhibitors. The medium was replaced with a serum-free medium containing CCK-8 reagent, followed by incubation at 37 °C for 2 h in the dark and the absorbance at 450 nm was measured. And the following inhibitors (all from MedChemExpress, USA) were used in this study: ferrostatin-1 (HY-100579), Z-VAD (3HY-16658B), chloroquine (HY-17589A), and necrostatin-1 (HY-15760).

### Measurement of ATP content

ATP content was performed by the ATP assay kit (S0026, Beyotime Biotechnology, China) according to the manufacturer’s instruction. Briefly, samples were lysed with ATP lysis buffer and measured by a luciferase reaction in which 560 nm light was emitted when D-luciferin was converted to oxyluciferin. Luminescence was measured using a luminometer (Promega GloMax® 20/20, Turner BioSytems Instrument, CA). ATP level was calculated according to a calibration curve with standard ATP samples. The total amount of protein was used for normalization to ATP contents.

### Measurement of mitochondrial oxygen consumption rate (OCR)

Measurement of OCR was performed at 37 °C in a high-resolution oxygraph (Oxygraph-2kOroboros Instruments, Innsbruck, Austria). NMCMs were placed in a 2 mL chamber at a final concentration of 1 × 10^6^ cells/mL. Then, mitochondrial poisons (1.5 μM oligomycin, 2 μM FCCP, 0.5 μM rotenone and 0.5 μM antimycin A) were added at the indicated time points. OCR was analyzed by DatLab6 software.

### Fatty acid oxidation (FAO) assay

An FAO kit (ab217602, Abcam, UK) combined with an extracellular oxygen consumption kit (ab197243, Abcam, UK) was used to detect the level of FAO in NMCMs according to the manufacturer’s instructions. Briefly, NMCMs were seeded in a 96-well plate at a density of 6 × 10^4^ cells/well in 200 µL of culture medium. Then, the corresponding reagents were added to the wells according to the kit protocols. Insert the prepared plate into a fluorescence plate reader pre-set to 37 °C. FAO levels were measured at 2 min intervals for 60 min using the following excitation and emission wavelengths: Ex/Em=380/650 nm.

### Mitochondrial membrane potential measurement

Mitochondrial membrane potential was assessed using the fluorescent potentiometric dye tetramethylrhodamine, ethyl ester (TMRE) (C2001S, Beyotime Biotechnology, China). Briefly, cells were seeded in a 24-well plate and allowed to adhere overnight. Prior to staining, the culture medium was removed, and cells were gently washed twice with pre-warmed PBS. Subsequently, cells were incubated with 100 nM TMRE in serum-free culture medium for 30 min. at 37 °C in a 5% CO₂ incubator, protected from light. Following incubation, the cells were washed three times with PBS to remove excess dye. Fluorescence images were acquired using Leica inverted microscopy (MATEO FL RUO) with a 550 nm excitation laser and a 575 nm emission filter. For quantitative analysis, the mean fluorescence intensity of TMRE in at least three wells per group was quantified per frame using Image J software.

### Malondialdehyde (MDA) determination

The intracellular MDA levels were measured using MDA Assay Kit (S0131S, Beyotime Biotechnology, China) in accordance with the manufacturer’s protocol. Briefly, NMCMs were lysed with lysis buffer and centrifuged at 13,500 rpm for 15 min. 100 µL supernatant was mixed with 200 µL MDA detection solution and placed in boiling water for 15 min. After cooling to room temperature, the sample was centrifuged at 1000 × g for 10 min. Then, 200 µL of the supernatant was resuspended into a 96-well plate and measured using a microplate reader at 532 nm.

### Measurement of 4-hydroxynonenal (4-HNE)

The intracellular 4-HNE levels were measured using enzyme-linked immunosorbent assay kit (JL46304, Jianglai Biotech, China). Briefly, samples and serially diluted standards (40 ng/mL to 0.625 ng/mL prepared in Universal Diluent) were added to 96-wells plates. Subsequently, biotinylated anti-4-HNE antibody (diluted 1:100 from 100× concentration) and then streptavidin-HRP conjugate (diluted 1:100 from 100× concentration) were added sequentially. All incubation steps were performed at 37 °C (30 min for samples/standards/antibody, 30 min for conjugate) with thorough washing between additions. After a final wash, TMB substrate was added and incubated for 15 min at 37 °C in the dark. The reaction was stopped with Stop Solution. Absorbance was measured at 450 nm using a microplate reader.

### Measurement of glutathione/glutathione disulfide (GSH/GSSG)

The contents of GSH/GSSG in NMCMs were measured as described in the steps supplied with the GSH and GSSG Assay Kit (S0053, Beyotime Biotechnology, China). Briefly, NMCMs were harvested and dissolved in protein-removing reagent M. Then, the samples were subjected to rapid freeze-thaw cycles using liquid nitrogen and thawed at 37 °C in a water bath. Supernatant was collected and added the diluted GSH clearance auxiliary solution and GSH removal solution, mixed immediately, and continued to react for 60 min at 25 °C. After the removal of GSH, GR and 5,5′-dithiobis (2-nitrobenzoic acid) were added in the reaction system and kept at room temperature for 5 min, and then the chromogenic reaction was started by adding NADPH for 20 min. Absorbance was analyzed using a microplate reader at 412 nm. GSSG level was quantified by the standard curve and divided by protein concentration. The GSH content was calculated according to the following formula: GSH=Total Glutathione-GSSG×2.

### Measurement of lipid peroxidation

The Image-iT® Lipid Peroxidation Kit (C10445, Life Technologies Corporation, USA), was used to detect the intracellular lipid peroxidation level. Briefly, cells were seeded in 24-well plates and incubated them overnight at 37 °C. Add Image-iT® Lipid Peroxidation Sensor (Component A) at a final concentration of 10 µM to the cells and incubated for another 30 min at 37 °C. Cells were washed with PBS and stained with Hoechst33342 (C1022, Beyotime Biotechnology, China) for 10 min. Images were captured by Leica inverted microscopy and reading the fluorescence at to separate wavelengths; one at excitation/emission of 581/591 nm for the reduced dye, and the other at excitation/emission of 488/510 nm for the oxidized dye. Fluorescence images from three wells per group were subsequently acquired and analyzed quantitatively using Image J software.

### Mitochondrial reactive oxygen species (ROS) assay

Mitochondrial ROS was stained with the MitoSOX™ Red Mitochondrial Superoxide Indicator (M36005, Invitrogen, USA). H9c2 cells were incubated at 37 °C with 5 µM MitoSOX working solution for 20 min. Then, the cells were washed with warm DMEM and stained with Hoechst33342 for 10 min. Images were captured by imaging microscope (Life EVOS® FL, USA). Firstly, 4 to 5 fluorescence images were taken from each well. The fluorescence intensity was statistically analyzed using ImageJ software and the average value was taken. Fluorescence images were subsequently acquired from five wells per group for quantitative statistical analysis.

### Measurement of intracellular ferrous iron

Intracellular Ferrous iron levels were measured using FerroOrange (F374, Dojindo, China). Briefly, cells were seeded in a 24-well plate and incubated overnight. Then, plates were washed in HBSS and were stained at 37 °C with 1 μM FerroOrange working solution for 30 min. Cells were washed with HBSS and stained with Hoechst33342 for 10 min. Following washes with HBSS, images were captured by Leica inverted microscopy. Subsequently, fluorescence images from three wells per group were acquired and subjected to quantitative statistical analysis with Image J software.

### Co-immunoprecipitation (Co-IP)

HEK293 or H9c2 cells were lysed with ice-cold IP lysis buffer (P0013, Beyotime Biotechnology, China). After centrifugation at 12,000 × g for 15 min at 4 °C, the supernatants were collected and incubated with an anti-His antibody (66005-1-Ig, Proteintech, China), anti-Flag antibody (80801-2-RR, Proteintech, China) or anti-DECR1 (sc-393473, Santa Cruz Biotechnology, CA) and Protein A/G beads (HY-K0202, MedChemExpress, USA) at 4 °C overnight. The beads were then washed, and the immunoprecipitated proteins were eluted and analyzed by SDS-PAGE.

### Quantitative proteomic analysis by LC-MS/MS

Mouse heart tissue samples were processed for mass spectrometry (MS)-based proteomic analysis by PTM Biolabs (Hangzhou, China). Briefly, tissues were lysed in a buffer containing 8 M urea and 1% protease inhibitor cocktail. The extracted proteins were then digested with trypsin to generate peptides. Subsequently, the peptides were desalted using a Strata X C18 column (Phenomenex) and dried by vacuum centrifugation. For in-depth analysis, the peptide mixture was fractionated by high-pH reverse-phase high-performance liquid chromatography (HPLC) on an Agilent 300Extend C18 column (5 μm particles, 4.6 mm × 250 mm). The collected fractions were further separated and purified by HPLC prior to MS analysis. Peptides were ionized using a Nanospray Ionization source and analyzed with an Orbitrap Exploris™ 480 mass spectrometer. For eukaryotic protein subcellular localization prediction, the WoLF PSORT tool was employed. For prokaryotic species, predictions were performed using CELLO. Functional domain analysis of differentially expressed proteins was conducted using PfamScan, while PSORTb (version 3.0) was used for subcellular localization visualization. Gene Set Enrichment Analysis (GSEA) was performed using RStudio (version 3.4.2). Detailed results are presented in Original Data File 1.

### Quantitative lipidomic analysis

Lipidomic analysis was conducted by LipidALL Technologies Co., Ltd. (Hangzhou, China). Lipids were extracted from approximately 45 mg of mouse heart tissues or approximately 5×10^4^ H9c2 cells using a modified version of the Bligh and Dyer’s method as described previously [51]. Briefly, tissues were homogenized in 900 µL of chloroform: methanol: Milli-Q H_2_O (3:6:1) (v/v/v). The homogenate was then incubated at 1500 rpm for 30 min at 4 °C. Subsequently, 350 µL of deionized water and 300 µL of chloroform were added to induce phase separation. Following centrifugation, the lower organic phase was collected. The extraction was repeated by adding 500 µL of chloroform to the remaining aqueous phase. The combined organic extracts were dried under a gentle nitrogen stream using a SpeedVac concentrator.

Lipidomic analysis was conducted at LipidALL Technologies using a Jasper HPLC coupled with Sciex TRIPLE QUAD 4500 MD as reported previously [52]. Separation of individual lipid classes by normal phase (NP)-HPLC was carried out using a TUP-HB silica column (i.d. 150 × 2.1 mm, 3 µm) with the following conditions: mobile phase A (chloroform: methanol:ammonium hydroxide, 89.5:10:0.5) and mobile phase B (chloroform:methanol:ammonium hydroxide:water, 55:39:0.5:5.5). The mass spectrometer was operated in multiple reaction monitoring (MRM) mode with electrospray ionization (ESI). Individual lipid species were quantified by referencing to appropriate internal standards. Relative lipid levels were determined based on the peak area ratio of each analyte to its corresponding internal standard. Detailed results are presented in Original Data File 2–4.

### Mass spectrometry analysis

Heart tissue lysates were prepared using IP lysis buffer supplemented with a protease inhibitor cocktail. Subsequently, the lysates were incubated overnight at 4^◦^C with anti-isotype-matched IgG or anti-GIPC1 antibodies along with Protein A/G Magnetic Beads. The immunoprecipitated complexes were collected, washed, and eluted according to the manufacturer’s protocol. The eluates were then separated by SDS-PAGE, and proteins were visualized by Coomassie blue staining (P0003S, Beyotime).

The mass spectrometry analysis was carried out at the Bioprofile Biotechnology Co., Ltd. (Shanghai, China). Briefly, protein bands of interest were excised and subjected to in-gel tryptic digestion. Gel pieces were destained by incubation with 800 µL of 0.1 M NH₄HCO₃ in 30% acetonitrile (ACN) until the Coomassie blue dye was completely removed. Proteins were then reduced and alkylated by sequential treatment with DTT and iodoacetamide in 100 mM NH₄HCO₃. After dehydration, the gel pieces were rehydrated and digested overnight at 37 °C with trypsin (20 ng/µL in 25-50 mM NH₄HCO₃). The resulting peptides were extracted, dried under vacuum, and reconstituted in 0.1% formic acid. Peptide cleanup and concentration were performed using C18 StageTip columns. Peptide separation was carried out using an Easy nLC 1200 nanoflow HPLC system (Thermo Scientific). The eluted peptides were analyzed by tandem mass spectrometry. The raw MS/MS data were processed using MaxQuant software (version 1.6.1.0) and searched against the UniProt protein database. For protein identification, false discovery rates (FDR) were set at ≤ 0.01 at both the peptide-spectrum match (PSM) and protein levels. All mass spectrometry data are provided in Supplementary Table 5.

### Molecular docking

The full-length structural models of GIPC1 and DECR1 were retrieved as initial predictions from the AlphaFold database (https://alphafold.ebi.ac.uk/). To refine these models, implicit solvent molecular dynamics (MD) simulations were performed, focusing on the relaxation of low-confidence regions identified by AlphaFold. The resulting MD-equilibrated structures were then used as input for global protein-protein docking using the ZDOCK program to predict the initial binding pose between GIPC1 and DECR1. The stability of the complex during MD simulation was assessed by calculating the root mean square deviation (RMSD) of the protein backbone atoms. Additionally, the binding free energy of the interaction was estimated from the MD trajectory.

### Surface plasmon resonance (SPR) assay

Protein-protein interaction analysis between recombinant human GIPC1 and DECR1 was performed on a Biacore T200 instrument (Cytiva, USA) at 25 °C. Briefly, the recombinant human GIPC1 protein (ab96769, Abcam) was diluted to 20 μg/mL in 10 mM sodium acetate buffer (pH 5.0) and immobilized on the CM5 chip surface via standard amine-coupling chemistry to achieve a target response level of approximately 5,000 response units (RU). Subsequent to this, serial dilutions of recombinant human DECR1 protein (ab105597, Abcam) were prepared in running buffer and injected over the immobilized GIPC1 surface at a flow rate of 30 μL/min. All buffers were prepared with ultrapure water and analytical-grade reagents. Sensorgram data were processed and analyzed using the Biacore T200 Evaluation Software. Double-referenced curves were aligned to the injection start point, and binding kinetics were calculated by globally fitting the data to a 1:1 Langmuir binding model.

### Statistical analysis

Statistical analysis was performed using GraphPad Prism software version 10.0 (GraphPad Software). Data were presented as mean ± SEM. Statistical significance was assessed using the paired Student’s t-test and one-way analysis of variance (ANOVA) followed by Tukey’s post-hoc-test. *P* value  <  0.05 was considered statistically significant.

## Supplementary information

Original Data1

Original Data2

Original Data3

Original Data4

Supplementary information

Original blots

checklist

## Data Availability

All data generated or analyzed during this study are included in this published article and its Supplementary Information files. Additional supporting data are available from the corresponding authors upon reasonable request.
