# Supplementary material for: GIPC1 governed ferroptosis by regulating DECR1-modulating lipid homeostasis during dilated cardiomyopathy (DCM)
Source: Cell Death Differ. 2026 Mar 5;33(8):1536–51. doi: 10.1038/s41418-026-01679-9 (PMC13434828; doi:10.1038/s41418-026-01679-9)
Supplement: Supplementary file 2 [file 41418_2026_1679_MOESM5_ESM.docx]

**Supplementary information**

**GIPC1 governed ferroptosis by regulating DECR1-modulating lipid homeostasis during dilated cardiomyopathy (DCM)**

**Running title:** GIPC1 protected dilated cardiomyopathy (DCM) against ferroptosis

Nannan Tang^1☨^, Ruxue Mu^1☨^, He Wang^1☨^, Jiaying Wu^1☨^, Jie Zhang^1^, Di Huang^2^, Yannan Han^1^, Wenjian Li^3^, Yuqing Chen^1^, Xiang Li^1^, Yilin Sun^1^, Zifeng Zhang^1^, Jinlu Zuo^1^, Ying Hu^1^, Yanan Yin^1^, Yang Qu^1^, Jinping Liu^4^, Lei Jiao^1^, Xue Liu^1^, Haihai Liang^1^, Ning Wang^1^, Yunlong Bai^1,5^, Yan Liu^6^, Bin Wang^4*^, Dan Zhao^2*^, Yu Liu ^1*^ and Baofeng Yang ^1*^

^☨^These authors contributed equally to this work.

^1^State Key Laboratory of Frigid Zone Cardiovascular Diseases (SKLFZCD), Department of Pharmacology (State Key Labratoray-Province Key Laboratories of Biomedicine-Pharmaceutics of China, Key Laboratory of Cardiovascular Research, Ministry of Education), College of Pharmacy, Harbin Medical University, Heilongjiang, 150081, PR China

^2^Department of Clinical Pharmacy, The Second Affiliated Hospital, Harbin Medical University, Heilongjiang, 150081, PR China

^3^Research Center for Pharmacoinformatics, College of Pharmacy, Harbin Medical University, Heilongjiang, 150081, PR China

^4^Department of Cardiovascular Ultrasound, Zhongnan Hospital of Wuhan University, Wuhan University, Wuhan, 430071, PR China

^5^Key Laboratory of Chronic disease Prevention and Control of Traditional Chinese Medicine of Guangdong Higher Education Institutes, School of Phammaceutical Sciences, Guangzhou University of Chinese Medicine, Guangzhou 510006, China

^6^College of Pharmacy, Hainan Medical University, Haikou, 571199, PR China

Correspondence to: Yu Liu, MD, PhD (E-mail Address: hmu_ly@163.com), Baofeng Yang, MD, PhD (E-mail Address: [yangbf@ems.hrbmu.edu.cn](mailto:yangbf@ems.hrbmu.edu.cn)), Dan Zhao, MD, PhD (E-mail Address: [zhaodan7719@163.com),](mailto:zhaodan7719@163.com),) or Bin Wang, MD, PhD (E-mail Address: [wangbin87098429@126.com)](mailto:zhaodan7719@163.com),); Department of Pharmacology, Harbin Medical University 157 Baojian Road, Harbin, Heilongjiang 150081, China; Tel: 86-451-86671354, Fax: 86-451-86675769

**Supplementary Figures**

**Supplementary Fig.1 GIPC1 cKO mice exhibited cardiac dysfunction and structure remodeling.**

(A-F) Representative echocardiographic images and corresponding measurements of EF, FS, IVSs, LVPWs and LVIDs of WT group and GIPC1 cKO mice group. *n* = 8 per group. (G-H) Masson staining images in histological sections with the area of fibrosis (blue) in the left ventricle quantified of the hearts of WT group and GIPC1 cKO mice group. Yellow arrow: fibrosis. *n* = 5 per group. scale=50 µm. (I-K) Real-Time PCR analysis of mRNA levels of β-MHC, ANP and BNP genes from the heart tissue of GIPC1 cKO and WT group. *n* = 8 per group. All quantitative data were expressed as mean±SEM, and statistical significance was analyzed using t-test, **P*<0.05, ***P*<0.01, ****P*<0.001.

**Supplementary Fig.2 Knockdown of GIPC1 regulated the incorporation of phospholipids.**

(A) The heatmap showing the statistical significance of PG-FA. (B) The heatmap showing the statistical significance of PC-FA. (C) The heatmap showing the statistical significance of PC-O-FA. (D) The heatmap showing the statistical significance of PI-FA. (B) The heatmap showing the statistical significance of PS-FA. *n* = 3 per group. **P*<0.05 *vs* WT.

**Supplementary Fig.3 DOX inhibited the interaction between GIPC1 and DECR1.**

(A) Top 10 binding poses between GIPC1 and DECR1 predicted by ZDOCK server. (B) The docking scores of the top 10 poses. (C) The first two poses (pose 1 and pose 2) were selected for molecular dynamics (MD) simulations, and the root mean square deviation (RMSD) of the GIPC1 and DECR1 complex and the binding interface were reported. Pose 1 was remarkably stable as evidenced by small complex RMSD values and interface RMSD values. (D) Atom densities of pose 1 and 2 projected onto a 2D plane. The highly density points of pose 1 highlighted its stability. (E) Binding free energy between GIPC1 and DECR1 in pose 1 and 2. The pose 1 binding mode is energetically favourable with around -50 kcal/mol binding free energy.

**Supplementary Tables**

**Supplementary Table 1. The sequences of plasmids.**

| **Name** | **Sequence** | **Vendor or Source** |
| --- | --- | --- |
| GIPC1 | Froward: CGCAAATGGGCGGTAGGCGTG  Reverse: TTATTAGGAAAGGACAGTGGG | Genechem, China |
| DECR1 | Froward: GTGGATCCGAGCTCGGTACCCGCCACCATGGCGCTGCTGGGCCGTGC  Reverse: GAGCGTATGTTAGTACTATCG | Genechem, China |

All the genes correspond to *mus musculus.*

| **Name** | **Sequence** | **Vendor or Source** |
| --- | --- | --- |
| GIPC1 | Froward: CGCAAATGGGCGGTAGGCGTG  Reverse: CTACCGGTGGATGTGGAATG | Hanyin, China |
| DECR1 | Froward: CAAGCTGTGACCGGCGCCTTACGAATTCGCCACCATGAAGCTACCGGCCA  Reverse: ACCCCATCGATGGACCGGTCGGGATCCTTAATGGTGATGGTGATGATGG | Hanbio, China |

All the genes correspond to *human*

**Supplementary Table 2. The sequences of siRNAs.**

| **Name** | **Sequence** | **Vendor or Source** |
| --- | --- | --- |
| NC | Froward: UUCUCCGAACGUGUCACGUTT  Reverse: CGCAAATGGGCGGTAGGCGTG | Genepharma, China |
| GIPC1 | Froward: GCAGUGUGAUUGACCACAUTT  Reverse: AUGUGGUCAAUCACACUGCTT | Genepharma, China |

All the genes correspond to *mus musculus.*

**Supplementary Table 3. Antibodies used for Western blot.**

| **Antibodies** | Company | **Catalog #** |
| --- | --- | --- |
| GIPC1 | Proteintech, China | 14822-1-AP |
| DECR1 | ABclonal, China | A13014 |
| β-MHC | Proteintech, China | 22280-1-AP |
| PTGS2 | Cell Signaling Technology, USA | 12282S |
| SLC7A11 | Abcam, UK | ab307601 |
| GPX4 | Abcam, UK | ab125066 |
| ACSL4 | Abways, USA | DY1198 |
| GAPDH | Proteintech, China | 60004-1-Ig |
| VDAC1 | Proteintech, China | 10866-1-AP |
| α-Tubulin | Proteintech, China | 11224-1-AP |
| Flag | Cell Signaling Technology, USA | 14793S |
| His | Proteintech, China | 51064-2-AP |
| IRDye-800 secondary goat anti-Mouse | LI-COR Biosciences, USA | 926-32210 |
| IRDye-800 secondary goat anti-Rabbit | LI-COR Biosciences, USA | 926-32211 |

**Supplementary Table 4. Sequences of Primers used for Real-Time PCR.**

| **Primers** | **Sequence** |
| --- | --- |
| GIPC1 | Froward: 5’-GGAGCTGTACGGCAAGATCG-3’  Reverse:5’- CTTGATGCGCTTAATGAAGGC-3’ |
| β-MHC | Froward: 5’-TTACTTGCTACCCTCAGGTGG-3’  Reverse: 5’-CTCCTTCTCAGACTTCCGCA-3’ |
| ANP | Froward: 5’-GTGCGGTGTCCAACACAGAT-3’  Reverse: 5’-CTCCTTCTCAGACTTCCGCA-3’ |
| BNP | Froward: 5’-GAGGTCACTCCTATCCTCTGG-3’  Reverse: 5’-GCCATTTCCTCCGACTTTTCTC-3’ |
| Cpt1 | Froward: 5’-GTGCTGGAGGTGGCT TTGG-3’  Reverse: 5’-CAGTGCTTGACGGATGTGG-3’ |
| Cpt2 | Froward: 5’-CAGCACAGCATCGTACCCA-3’  Reverse: 5’-TCCCAATGCCGTTCTCAAAAT-3’ |
| Acat1 | Froward: 5’-CAGGAAGTAAGATGCCTGGAAC-3’  Reverse: 5’-TGCAGCAGTACCAAGTTTAGTG-3’ |
| Acadm | Froward: 5’-AACACAACACTCGAAAGCGG-3’  Reverse: 5’-TTCTGCTGTTCCGTCAACTCA-3’ |
| Acaa2 | Froward: 5’-CTGCTACGAGGTGTGTTCATC-3’  Reverse: 5’-AGCTCTGCATGACATTGCCC-3’ |
| GAPDH | Froward: 5’-GATGCCCCCATGTTTGTGAT-3’  Reverse: 5’-GGCATGGACTGTGGTCATGAG-3’ |

All primers correspond to *mus musculus.*

**Supplementary Table 5. Mass spectrometry identification.**

| **Proteins** | **Peptides** | **Razor + unique peptides** | **Unique peptides** | **Sequence coverage [%]** | **Score** | **Sequence coverage 2 [%]** | **Sequence**  **coverage**  **4 [%]** | **Sequence**  **coverage 5 [%]** | | **Sequence coverage 6 [%]** |
| --- | --- | --- | --- | --- | --- | --- | --- | --- | --- | --- |
| Gfi1 | 1 | 1 | 1 | 23.9 | 3.2237 | 23.9 | 23.9 | 23.9 | | 0 |
| Atp5e | 1 | 1 | 1 | 15.4 | 1.151 | 15.4 | 0 | 0 | | 0 |
| Ddt;Gm20441 | 1 | 1 | 1 | 12.7 | -2 | 12.7 | 0 | 0 | | 0 |
| Anxa5 | 3 | 3 | 3 | 10.7 | 4.3897 | 10.7 | 0 | 0 | | 0 |
| Msrb3 | 1 | 1 | 1 | 10.5 | -2 | 10.5 | 0 | 0 | | 0 |
| Atp5d | 1 | 1 | 1 | 8.3 | 24.077 | 8.3 | 0 | 0 | | 0 |
| Rmdn1 | 2 | 2 | 2 | 6.6 | 1.6372 | 6.6 | 0 | 0 | | 3.6 |
| Rpl5 | 1 | 1 | 1 | 6.1 | 8.1314 | 6.1 | 0 | 0 | | 6.1 |
| Oxnad1 | 1 | 1 | 1 | 5.1 | 1.7961 | 5.1 | 5.1 | 0 | | 0 |
| Coq3 | 1 | 1 | 1 | 4.6 | 1.7272 | 4.6 | 0 | 0 | | 0 |
| Decr1 | 2 | 2 | 2 | 8.1 | 2.129 | 4.2 | 3.9 | 0 | | 4.2 |
| Psmc6 | 1 | 1 | 1 | 3.6 | 1.1202 | 3.6 | 0 | 0 | | 0 |
| Acad9 | 1 | 1 | 1 | 2.4 | 1.6883 | 2.4 | 2.4 | 0 | | 2.4 |
| Prickle1 | 1 | 1 | 1 | 2 | 1.205 | 2 | 0 | 0 | | 0 |
| Hgs | 1 | 1 | 1 | 1.4 | 2.1004 | 1.4 | 0 | 1.4 | | 0 |
| Gm17673 | 1 | 1 | 1 | 1.3 | -2 | 1.3 | 1.3 | 0 | | 0 |
| Zmym1 | 1 | 1 | 1 | 1.2 | -2 | 1.2 | 0 | 0 | | 0 |
| Abcb4 | 1 | 1 | 1 | 0.6 | -2 | 0.6 | 0 | | 0 | 0 |
| Kntc1 | 1 | 1 | 1 | 0.5 | -2 | 0.5 | 0.5 | | 0 | 0 |
| Trio;Kalrn | 1 | 1 | 1 | 0.4 | -2 | 0.4 | 0 | | 0 | 0 |
