# Supplementary material for: GIPC1 governed ferroptosis by regulating DECR1-modulating lipid homeostasis during dilated cardiomyopathy (DCM)
Source: Cell Death Differ. 2026 Mar 5;33(8):1536–51. doi: 10.1038/s41418-026-01679-9 (PMC13434828; doi:10.1038/s41418-026-01679-9)
Supplement: Supplementary file 3 [file 41418_2026_1679_MOESM6_ESM.docx]

**GIPC1 governed ferroptosis by regulating DECR1-modulating lipid homeostasis during dilated cardiomyopathy (DCM)**

**Figure 1C**

**Figure 1O**

**Figure 1R**

**Figure 1U**

**Figure 2B**

**Figure 2L**

**Figure 2S**

**Figure 4K**

**Figure 4L**

**Figure 4W**

**Figure 4X**

**Figure 5I**

**Figure 6D**

**Figure 6E**

**Figure 6G**

**Figure 7 A**

**Figure 7 J**
